# Mitochondrial dysfunction-mediated metabolic remodeling of TCA cycle promotes Parkinson’s disease through inhibition of H3K4me3 demethylation

**DOI:** 10.1038/s41420-025-02651-1

**Published:** 2025-07-29

**Authors:** Xiaoyuan Zhang, Fali Zhang, Yue Zeng, Aiying Li, Jiamao Yan, Pei Li, Kexin Qin, Teng Zhang, Jiaojiao Huang, Minghui Zhao, Massimo De Felici, Yang Zhou, Wei Shen

**Affiliations:** 1 College of Animal Science and Technology, Qingdao Agricultural University, Qingdao, China; 2 State Key Laboratory of Reproductive Regulation and Breeding of Grassland Livestock (R2BGL), College of Life Sciences, Inner Mongolia University, Hohhot, China; 3 Department of Biomedicine and Prevention, University of Rome Tor Vergata, Rome, Italy

**Keywords:** Parkinson's disease, Pathogenesis

## Abstract

Parkinson’s disease (PD), a neurodegenerative disorder caused by complex factors, is usually associated to mitochondrial dysfunctions but the links between such disorder and PD remain object of research. Here, we report that impaired mitochondrial quality control (MQC) system is a molecular basis of the mitochondrial dysfunction in PD and that tricarboxylic acid cycle (TCA cycle) disorder is the main feature of such mitochondrial dysfunction. Multi-omics analysis revealed that MDH2, OGDHL and IDH3G enzymes are bottlenecks in the enzymatic reactions of the TCA cycle in PD. Mechanistically, the abnormal α-KG/fumarate ratio caused by the TCA cycle bottleneck inhibits histone H3K4me3 demethylation and further enhances the expression of alpha-synuclein (SNCA), which may promote PD at an early stage. On these bases, we proposed a number of PD therapeutic strategies targeting mitochondria and histone methylation modifications, which proved to be effective in in vitro or in vivo models, especially citrate supplementation, in restoring normal TCA cycle enzymatic reactions. Taken together, our work highlights the non-negligible regulatory role of “mitochondrial-nuclear” communication in PD and provides important insights for the development of PD therapeutic strategies.

## Introduction

Parkinson’s disease (PD) is a progressive neurodegenerative disease characterized by the loss of dopaminergic neurons and the accumulation of alpha-synuclein (alpha-syn, SNCA) in the substantia nigra (SN) [1, 2]. Although the specific molecular mechanisms that lead to the pathophysiology of PD remain unclear, a large number of evidences suggest that mitochondrial dysfunction is highly associated with such disease [3, 4].

Mitochondrial quality control (MQC) system is a conservative mechanism for maintaining the mitochondrial functions of eukaryotic cells, which mainly includes three aspects: mitochondrial biogenesis, dynamics and autophagy [5]. MQC system regulates mitochondrial homeostasis to adapt mitochondria to the local energy supply and overall metabolic needs of cells. The brain is highly sensitive to changes in energy metabolism, and there have been many reports that abnormalities in MQC system lead to neurodegenerative diseases [6–8]. Interventions targeting mitochondria, especially MQC pathways, such as SRT1720, curcumin, and Q14 peptide have shown extraordinary potential in laboratory tests or clinical applications for the treatment of neurodegenerative diseases [9–12].

The tricarboxylic acid cycle (TCA cycle), also known as the Krebs cycle, is the main pathway for mitochondrial energy production and metabolic conversion [13, 14]. Although multiple reports have pointed out the association between the TCA cycle and PD, the molecular details of how the TCA cycle regulates the pathogenesis of PD remain unclear. Many metabolites in the TCA cycle can participate in the regulation of epigenetic modifications, and they are often designated as substrates or activity regulators of certain epigenetic modification enzymes [15]. α-KG is a required substrate of the Jumonji C domain containing lysine demethylases (KDM2-7) [15] and fumarate is considered an inhibitor of KDMs activity [16]. It is still unclear how these metabolites regulate histone modifications and cell fate during PD.

Here, we investigated the association of PD with mitochondrial dysfunction and epigenetic modifications dependent on TCA cycle metabolites. Using in vitro and in vivo models, we confirmed that the MQC system is impaired in PD. TCA cycle disorders caused by mitochondrial dysfunction are mainly manifested as an imbalance in the ratio of metabolic intermediates α-KG and fumarate. This metabolic remodeling hinders the demethylation of H3K4me3 and enhances SNCA expression by inhibiting the activity of KDMs, ultimately promoting PD. Our work suggests that targeting mitochondria and their related epigenetic modifications may be a promising intervention strategy for PD from three perspectives: activating the MQC system, correcting the TCA cycle, and restoring histone methylation levels.

## Results

### Bulk RNA-seq identifies transcriptional signatures of distinct striatal regions in PD

**Fig. 1:**
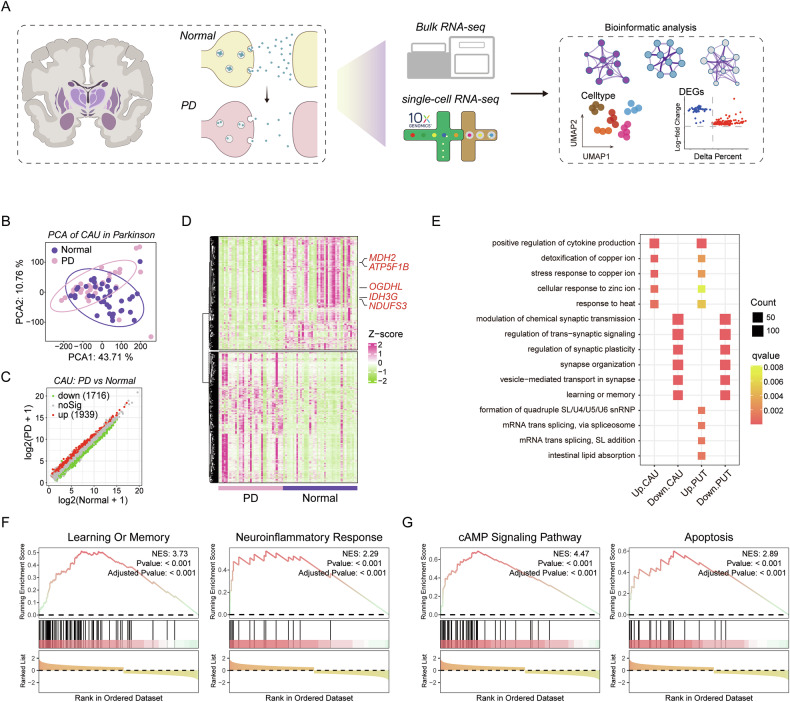
Transcriptional signatures of different striatal regions. **A** Schematic diagram of the joint analysis of multi-omics data from the normal group and PD group. Principal component analysis (**B**) and DEGs (**C**) of transcriptome data of CAU from normal group and PD group. **D** Heat map shows the expression patterns of DEGs of the transcriptome data of CAU in the normal group and PD group, and some genes of interest are listed on the right. **E** KEGG analysis of DEGs based on CAU and PUT transcriptome data. GSEA analysis of CAU transcriptome data based on the relevant GO term gene list (**F**) and KEGG pathway gene list (**G**).

We obtained bulk RNA-seq datasets of CAU and PUT structures in the striatum of PD patients and single-cell RNA-seq (scRNA-seq) datasets from the SN of PD patients from previous reports for joint analysis. Multi-omics analysis helped us to reveal the pathogenesis of PD more comprehensively (Fig. 1A). Analysis of DEGs in CAU and PUT samples showed that compared with the Normal group, 1716 and 1939 DEGs were downregulated and upregulated in CAU of PD group, respectively, and 1771 and 2463 DEGs were downregulated and upregulated in PUT of PD group, respectively (Figs. 1B, C and S1A, B). The expression patterns of multiple mitochondria-related genes in CAU or PUT of both normal and PD groups were unexpectedly similar. Notably, the expression of *MDH2*, *OGDHL*, *IDH3G*, *NDUFS3* and *ATP5F1B* genes was significantly reduced in the PD group (Figs. 1D and S1C). GO and KEGG analysis of DEGs showed that, similar to previous reports, the ion stress response of striatum was enhanced in the PD group, while processes such as synaptic organization, vesicle-mediated transport, and signal transmission were significantly inhibited in the PD group (Figs. 1E and S1D), reflecting severe synaptic damage [17]. Considering the pathological characteristics of PD, such as cognitive dysfunction [18], neuroinflammatory response [19], abnormal guanosine 3’,5’-monophosphates (cAMP and cGMP) signaling [20], and dopaminergic neuron loss [1], we performed gene set enrichment analysis (GSEA) on striatal DEGs using the relevant KEGG pathway or GO gene lists as the background gene set. Interestingly, GSEA analysis showed that apoptosis signaling, cAMP signaling pathway (KEGG pathway), learning and memory, and neuroinflammatory response (GO term) were upregulated in the PD striatum (Figs. 1F, G and S1E, F). It is particularly noteworthy that the expression patterns of these signals were surprisingly conserved among different regions of the striatum.

### Identification of mitochondrial dysfunction in PD

**Fig. 2:**
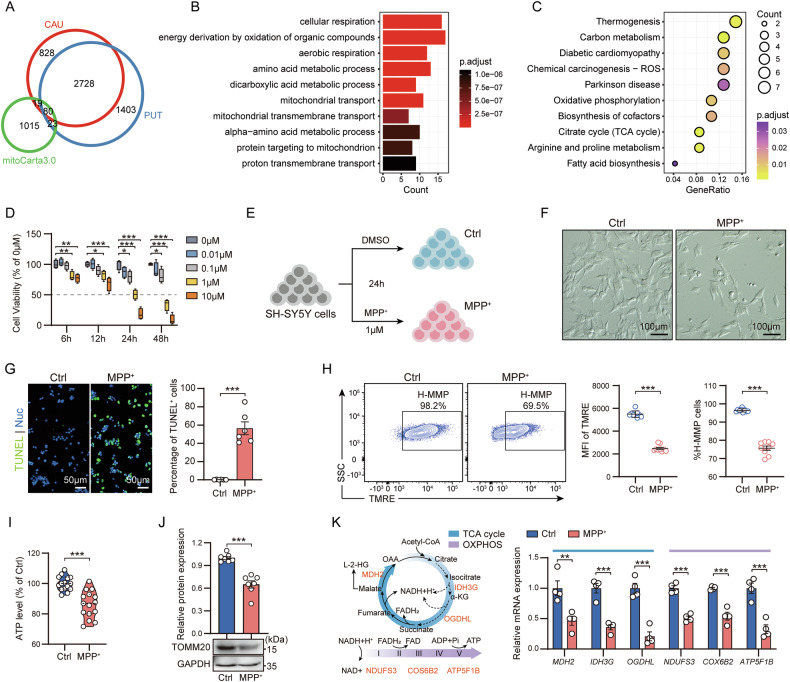
Mitochondrial dysfunction is a concomitant cellular injury phenotype in PD. **A** Venn diagram shows the overlapping genes among CAU DEGs, PUT DEGs and mitochondrial-related genes. KEGG analysis (**B**) and GO analysis (**C**) based on overlapping genes. **D** Cell viability analysis of SH-SY5Y cells treated with MPP^+^ at the indicated times and concentrations. Data are mean ± SEM; *n* = 4 biological replicates. **E** Schematic diagram of the establishment of the PD cell model. **F** Bright field images of cells in the PD cell model. **G** Representative images (left) and ratios (right) of TUNEL^+^ cells in PD cell model. Data are mean ± SEM; *n* = 6 biological replicates. **H** Representative images of flow cytometric analysis of MMP in PD cell model (left), median fluorescence intensity (MFI; middle), and the proportion of high MMP (H-MMP) to total cell number (right). Data are mean ± SEM; *n* = 9 biological replicates. **I** ATP levels in PD cell model. The upper and lower boundary in the plot indicates the upper and lower quantiles, the line inside the plot the median; *n* = 15 biological replicates. **J** TOMM20 protein expression level in PD cell model. Data are mean ± SEM; *n* = 7 biological replicates. **K** mRNA expression levels of TCA cycle and OXPHOS-related genes in PD cell model. Data are mean ± SEM; *n* = 4 biological replicates.

The conserved transcriptional signatures of CAU and PUT suggest a common driving mechanism underlying PD pathophysiology. Considering the significant changes in mitochondrial function in the pathophysiology of PD [13, 21], combined with the MitoCarta3.0 database, we conducted a comprehensive analysis of the differentially expressed gene sets from CAU and PUT. There were 122 genes that overlapped, and most of them (80 genes) were conserved DEGs in CAU and PUT (Fig. 2A). Enrichment analysis based on these DEGs suggested that mitochondrial functions, such as TCA cycle and oxidative phosphorylation (OXPHOS), were severely impaired in PD (Fig. 2B, C).

To further characterize the severity of mitochondrial dysfunction in PD, we established an MPP^+^-induced PD cell model using the neuroblastoma SH-SY5Y cell line (Fig. 2D, E). In the PD model, the degree of cell damage shows a significant time dose dependency effect (Fig. 2D). Compared with control (Ctrl), MPP^+^ treatment increased synaptic damage and caused high levels of apoptosis (Fig. 2F, G), which is consistent with the previous rich analysis results (Figs. 1E, G and S1D, F). OXPHOS uses mitochondrial membrane potential (MMP) to drive ATP production, so MMP and ATP levels are key indicators of OXPHOS. Our results showed that MMP and ATP levels were significantly reduced in PD cell model, reflecting impaired mitochondrial OXPHOS in PD (Fig. 2H, I). In addition, MPP^+^ treatment also reduced mitochondrial content (quantified using the mitochondrial outer membrane marker TOMM20) in the in vitro model (Fig. 2J). In order to more accurately evaluate mitochondrial function in PD, we further examined the expression levels of some TCA cycle enzymes and subunits related to the OXPHOS complex in the in vitro model and found that MPP^+^ treatment significantly reduced the expression of genes encoding TCA cycle enzymes and OXPHOS complex-related subunits (Fig. 2K). Thus, confirming severe mitochondrial dysfunction in PD.

### The activation of MQC system alleviates cell damage in PD cell model

**Fig. 3:**
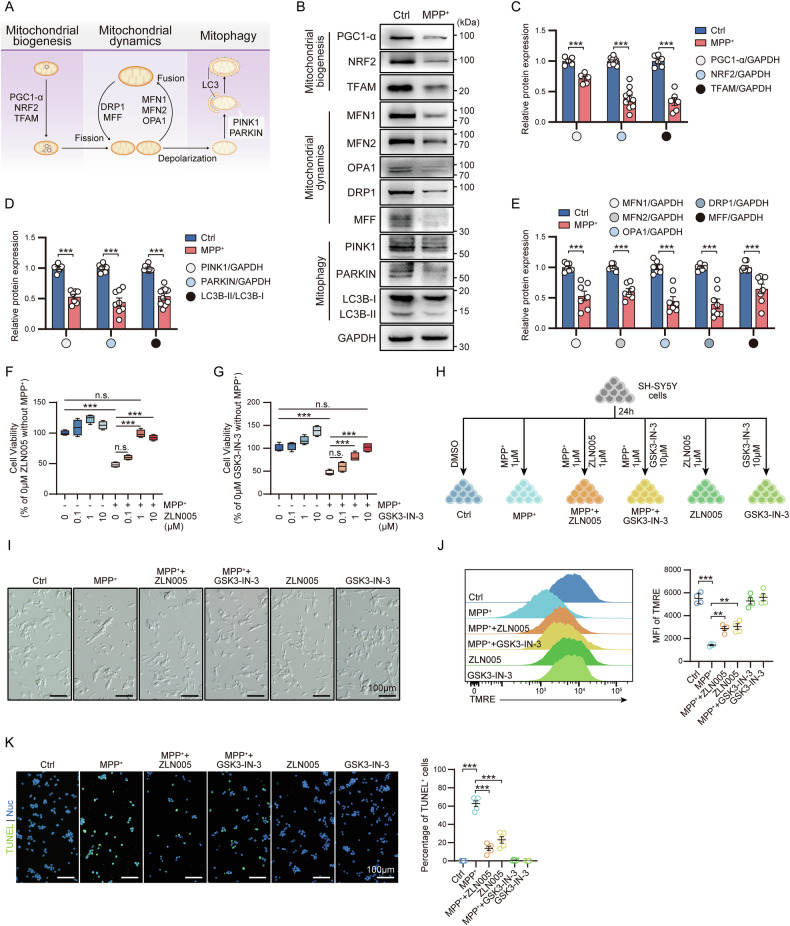
Targeting the MQC system restore mitochondrial function and alleviate cell damage in PD cell in vitro model. **A** Schematic diagram of the MQC system. Representative images (**B**) and expression level analysis of proteins related to the MQC system in PD cell model, including mitochondrial biogenesis (**C**), autophagy **(D**) and dynamics (**E**). Data are mean ± SEM; *n* = 5 to 10 biological replicates. **F** Cell viability analysis of PD cell model treated with ZLN005 at the indicated concentrations. Data are mean ± SEM; *n* = 4 biological replicates. **G** Cell viability analysis of PD cell model treated with GSK3-IN-3 at the indicated concentrations. Data are mean ± SEM; *n* = 4 biological replicates. **H** Schematic diagram of the establishment of the cell model treated with ZLN005 and GSK3-IN-3. **I** Bright field images of PD cell model treated with ZLN005 and GSK3-IN-3. **J** Representative images of flow cytometric analysis of MMP (left) and MFI (right) in PD cell model treated with ZLN005 and GSK3-IN-3. Data are mean ± SEM; *n* = 4 biological replicates. **K** Representative images (left) and ratios (right) of TUNEL^+^ cells in PD cell model treated with ZLN005 and GSK3-IN-3. Data are mean ± SEM; *n* = 5 biological replicates.

In mammalian cells, MQC system involving mitochondrial biogenesis, dynamics and autophagy, strictly regulates mitochondrial function [5]. The mitochondrial dysfunction found in the PD model prompted us to verify whether the MQC system plays a role in PD (Fig. 3A). Actually, compared with the Ctrl group, the expression of mitochondrial biogenesis-related proteins PGC1-α, NRF2, and TFAM was reduced in PD (Fig. 3B, C). In addition, the low-level expression of mitochondrial fusion-related proteins MFN1, MFN2, OPA1 and fission-related proteins DRP1 and MFF reflected the abnormal mitochondrial dynamics in PD, which reduced the turnover efficiency of mitochondria (Fig. 3B, D). The reduction in the expression of mitochondrial autophagy-related proteins PINK1 and PARKIN and the autophagic flux LC3B-II/LC3B-I also reflected the inefficient mitochondrial clearance in the PD model (Fig. 3B, E).

Taken together these last results, indicated that the MQC system was severely impaired in the MPP^+^-induced in vitro model suggesting that such defect is the major cause of the mitochondrial dysfunction in PD.

To clarify further the role of the MQC system in regulating mitochondrial functional homeostasis in PD, we used ZLN005, a mitochondrial biogenesis activator, and GSK-IN-3, a mitophagy inducer, to activate the MQC system and evaluate the effects of the two rescue strategies on mitochondrial function and cell damage in PD (Fig. 3F–H). The results showed that activation of mitochondrial biogenesis or autophagy restored the impaired cell viability in the PD model (Fig. 3F, G) and alleviated synaptic damage (Fig. 3I). In addition, activation of the MQC system enhanced the mitochondrial function of the PD cell model while reducing apoptosis level (Fig. 3J, K).

### scRNA-seq identified MDH2, OGDHL and IDH3G enzymes as bottleneck of the TCA cycle in PD

**Fig. 4:**
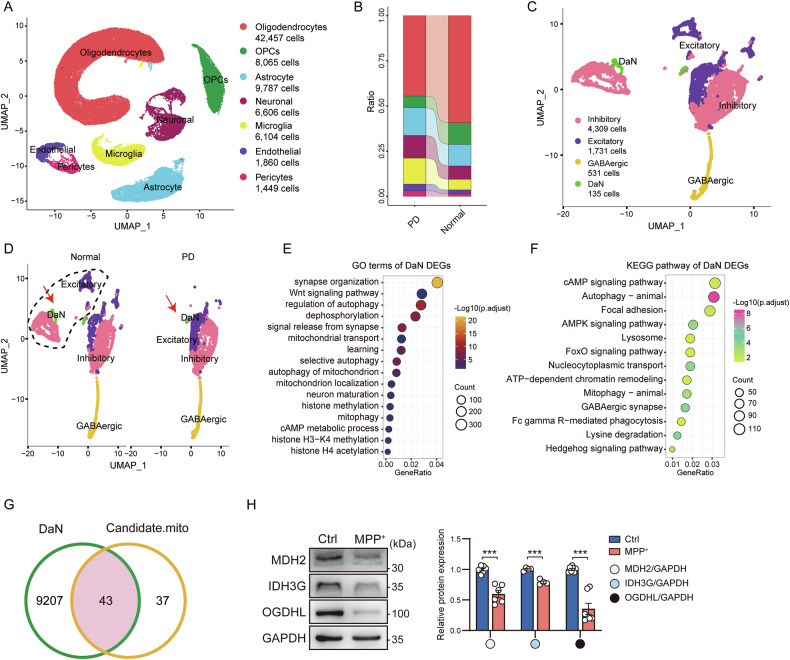
Multi-omics analysis identifies TCA cycle bottleneck in PD. **A** UMAP plot showing the main seven identified cell types. **B** Bar plot showing the ratio of each cluster in each group. **C** UMAP plot showing the four major neuronal subtypes. **D** UMAP distribution characteristics of each neuronal subtype in the normal group (left) and PD group (right). GO (**E**) and KEGG (**F**) analysis of DEGs in DaN. **G** Venn plots showing the overlapping genes between DaN DEGs and candidate mitochondrial gene sets in transcriptome data. **H** Representative images (left) and expression level analysis (right) of MDH2, IDH3G, and OGDHL protein expression in PD cell models. Data are mean ± SEM; *n* = 4–7 biological replicates.

To further explore the contribution of mitochondrial dysfunction to PD, we acquired single-cell transcriptome data from PD brains. We then used uniform manifold approximation and projection (UMAP) dimensionality reduction and cluster analysis to characterize seven major cell types: oligodendrocytes for high expression of *MOBP* and *MOG* genes, oligodendrocyte precursors (OPCs) for *VCAN*, astrocytes for *AQP4* and *GFAP*, neuronal for *GAD1* and *GAD2*, microglia for *CD74*, endothelial for *EGFL7* and *GLDN5*, and pericytes for *PDGFRB* (Figs. 4A and S2A, B). Next, we investigated whether different clusters varied between Normal and PD; the results showed that the proportion of oligodendrocytes and OPCs was decreased in the PD group, which may be related to altered myelination [22]. Conversely, the proportion of microglia was increased in the PD group (Figs. 4B and S2C), which reflects the aggravated neuroinflammation [23]. To explore neuronal lesions in PD, neuronal clusters from the scRNA-seq dataset were extracted for further analysis and SN neurons subdivided into four subtypes based on different cell-specific marker genes: dopamine (DaN), GABAergic, Inhibitory and Excitatory neurons (Figs. 4C and S2D). Figure S2E shows the top five DEGs of each neuronal subtype between the Normal and PD groups. In line with previous reports [24, 25], UMAP distribution characteristics highlighted that DaN and Excitatory neurons were significantly altered in PD (Fig. 4D). Moreover, GO and KEGG enrichment analysis of DaN-related DEGs showed that the processes of mitophagy, transport and localization were significantly affected in PD (Fig. 4E, F). Interestingly, terms or pathways such as histone modification and chromatin remodeling were also enriched, suggesting that some epigenetic modification processes were also affected in such PD neurons (Fig. 4E, F).

Finally, given the tissue heterogeneity in PD pathophysiology, we jointly analyzed the mitochondria-associated candidate gene set (containing 80 DEGs) from the bulk RNA-seq data of the striatum CAU and PUT with the DAN-associated DEGs from the midbrain scRNA-seq data. Joint analysis revealed 43 tissue-conserved genes (Fig. 4G and Supplementary Table 3), of which *MDH2* and *OGDHL*, which encode key metabolic enzymes of TCA cycle (Figs. 1D, 2K, 4H and S1C), were significantly downregulated in both PD transcriptome data and cell model. In addition, the expression of TCA cycle metabolic enzyme IDH3G was also significantly reduced in PD cell model, which was also included in the downregulated DEGs of striatal transcriptome data (Figs. 1D, 2K, 4H and S1C).

### TCA cycle dysregulation-mediated metabolic remodeling inhibits H3K4me3 demethylation in PD

**Fig. 5:**
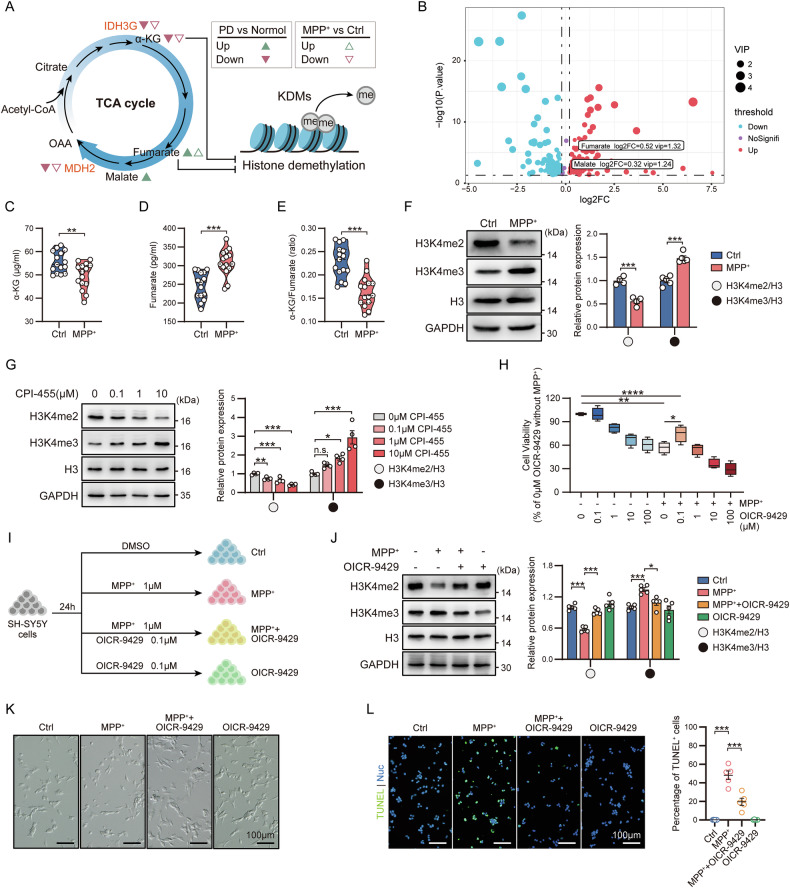
TCA cycle remodeling promotes PD by increasing H3K4me3 levels. **A** Schematic diagram of TCA cycle metabolites regulating histone demethylation. The changing trends of some metabolic enzymes or metabolites are derived from manual annotation of metabolomics data or analysis results of in vitro experiments. **B** Volcano plot showing serum differential metabolites between early and late stages of PD. α-KG level (**C**), fumarate level (**D**), and α-KG/fumarate ratio (**E**) in PD cell model. The upper and lower boundary in the plot indicates the upper and lower quantiles, the line inside the plot the median; *n* = 15 biological replicates. **F** Representative images (left) and expression level analysis (right) of H3K4me2 and H3K4me3 protein expression in PD cell model. Data are mean ± SEM; *n* ≥ 4 biological replicates. **G** Representative images (left) and expression level analysis (right) of H3K4me2 and H3K4me3 protein expression in PD cell model treated with different concentrations of CPI-455. Data are mean ± SEM; *n* = 4 biological replicates. **H** Cell viability analysis of PD cell model treated with OICR-9429 at the indicated concentrations. Data are mean ± SEM; *n* = 4 biological replicates. **I** Schematic diagram of the establishment of the OICR-9429-treated PD cell model. **J** Representative images (left) and expression level analysis (right) of H3K4me2 and H3K4me3 protein expression in OICR-9429-treated PD cell model. Data are mean ± SEM; *n* = 5 biological replicates. **K** Bright field images of OICR-9429-treated PD cell model. **L** Representative images (left) and ratios (right) of TUNEL^+^ cells in the OICR-9429-treated PD cell model. Data are mean ± SEM; *n* = 5 biological replicates.

Enzymatic reaction bottlenecks may cause extensive TCA cycle-related metabolic remodeling [13, 26]. We speculate that downregulation of IDH3G may lead to a decrease in the circulating levels of its enzymatic reaction products such as α-KG, while downregulation of MDH2 may lead to the accumulation of its enzymatic reaction substrates such as malate and fumarate (Fig. 5A). The results of the analysis of metabolome dataset from early and late PD patients were consistent with our speculation. As PD progressed, the level of α-KG in the serum of PD patients decreased, while the level of fumarate increased (Fig. 5A, B). We also detected similar changes in the PD cell model (Fig. 5A, C, D). Notably, this metabolic remodeling reduces the intracellular α-KG/fumarate ratio (Fig. 5A, E), which may affect the histone demethylation process by inhibiting the activity of KDMs [15]. Considering that the methylation process of histone H3K4 was enriched in the DaN-related DEGs of midbrain scRNA-seq data (Fig. 4E), we examined the expression levels of histone H3K4me2 and H3K4me3 in PD cell model. The results showed that the expression of H3K4me2 decreased in the PD cell model (Fig. 5F). On the contrary, the level of H3K4me3 increased significantly, indicating that the demethylation process of H3K4me3 was inhibited in PD (Fig. 5F). SH-SY5Y cells treated with CPI-455 (a KDMs inhibitor) showed inhibition of H3K4me3 demethylation consistent with the PD cell model (Fig. 5G). Based on these results, it is not difficult to determine that the TCA cycle enzymatic reaction bottleneck in PD inhibits the demethylation process of histone H3K4me3 by remodeling the metabolic state, and the reduction of the intracellular α-KG/Fumarate ratio is the main feature of this TCA cycle metabolic remodeling. Next, we used OICR-9429, an H3K4me3-specific histone methyltransferase inhibitor, to target and reduce the level of H3K4me3 to explore the regulatory role of histone methylation modification in PD. The results showed that OICR-9429 treatment reduced the level of H3K4me3 and enhanced cell viability in the PD cell model (Fig. 5H–J). At the same time, we also showed that reducing the level of histone H3K4me3 alleviated synaptic damage and reduced apoptosis in the PD cell model (Fig. 5K, L). In summary, we showed that metabolic remodeling mediated by TCA cycle dysregulation inhibits the demethylation of H3K4me3 in PD, and that targeting this epigenetic modification might be an effective intervention strategy for PD.

### Citrate exhibits neuroprotective effects by correcting the abnormal α-KG/Fumarate ratio in PD

**Fig. 6:**
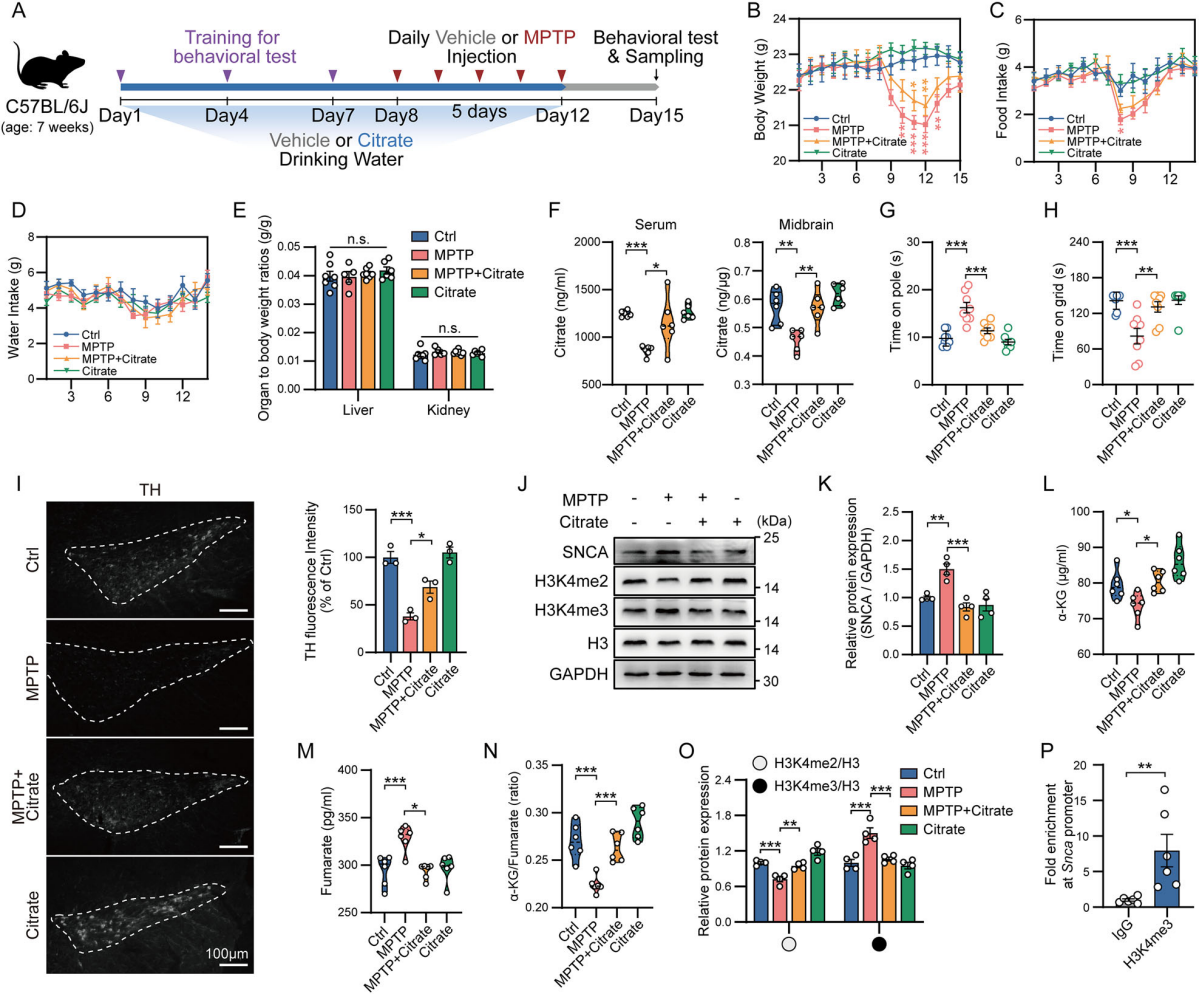
Citrate supplementation exerts neuroprotective effects in PD mice. **A** Schematic diagram of the construction of MPTP-treated and citrate-supplemented mouse model. Time change curves of body weight (**B**), food intake (**C**) and water intake (**D**) during the establishment of the MPTP-treated mice receiving treatment with citrate, * shows the significant difference between this group and the Ctrl group. Data are mean ± SEM; *n* ≥ 5 biological replicates. **E** Organ-to-body weight ratios in the MPTP-treated mice receiving treatment with citrate. Data are mean ± SEM; *n* ≥ 5 biological replicates. **F** Serum and midbrain citrate levels in MPTP-treated mice receiving citrate treatment. The upper and lower boundary in the plot indicates the upper and lower quantiles, the line inside the plot the median; *n* = 6 biological replicates. Motor activity in the pole test (**G**) and inverted grid test (**H**) for MPTP-treated mice receiving treatment with citrate. Data are mean ± SEM; *n* = 8 biological replicates. **I** Representative fluorescence images (left) and fluorescence intensity analysis (right) of TH protein expression in the SN of the MPTP-treated mice receiving treatment with citrate. Data are mean ± SEM; *n* = 3 biological replicates. **J** Representative images of SNCA, H3K4me2, and H3K4me3 protein expression in the MPTP-treated mice receiving treatment with citrate. **K** Expression level analysis of SNCA protein expression in the MPTP-treated mice receiving treatment with citrate. Data are mean ± SEM; *n* = 4 biological replicates. α-KG levels (**L**), fumarate levels (**M**), and α-KG/fumarate ratio (**N**) in the citrate-supplemented MPTP mouse model. The upper and lower boundary in the plot indicates the upper and lower quantiles, the line inside the plot the median; *n* = 6 biological replicates. **O** Expression level analysis of H3K4me2 and H3K4me3 protein expression in the MPTP-treated mice receiving treatment with citrate. Data are mean ± SEM; *n* = 4 biological replicates. **P** Detection of the enrichment level of the *Snca* promoter fragment bound to H3K4me3 using CUT&RUN-qPCR. Data are mean ± SEM; *n* = 6 biological replicates.

Based on the above results, we sought to find a natural supplement to correct the abnormal TCA cycle in PD. Given the extraordinary potential of citrate supplementation in improving cognitive ability, we constructed a subacute PD mouse model using MPTP and supplemented citrate through drinking water (Fig. 6A), which maximally simulated the dietary intake of natural supplements under physiological conditions [21]. Although continuous intraperitoneal injection of MPTP during model construction affected the food intake of mice, and this effect was reflected in changes in mouse body weight, the weight difference became no longer significant in the later stages of the model construction (Fig. 6B, C). During the establishment of the model, the water intake of mice was not affected, which ensured the stability of citrate supplementation (Fig. 6D). As expected, dietary citrate supplementation did not show systemic toxicity, while significantly increasing serum and midbrain citrate levels, confirming the safety and effectiveness of the citrate supplementation model, especially that citrate can cross the blood-brain barrier (BBB) and enter the midbrain (Fig. 6E, F). To evaluate the effect of citrate on motor dysfunction in the subacute PD model, we tested the motor ability and coordination ability of the model mice by behavioral tests. Compared with MPTP-treated mice, citrate supplementation reduced the time mice spent on the pole and increased the grasping time of mice on the inverted grid (Fig. 6G, H), indicating that citrate supplementation alleviated motor impairment in PD mice. Importantly, citrate supplementation restored the impaired tyrosine hydroxylase (TH; DaN marker) protein expression in the SN of PD mice and reduced MPTP-induced SNCA accumulation in the midbrain (Fig. 6I–K).

To further clarify the molecular details of citrate’s neuroprotective effects, we detected the changes in the abundance of α-KG and fumarate in the serum of PD mice after citrate supplementation. The results showed that citrate supplementation restored the abundance of α-KG and fumarate in PD mice to normal levels, and ultimately corrected the abnormal α-KG/Fumarate ratio (Fig. 6L-N). In addition, citrate supplementation also relieved the inhibitory effect of abnormal α-KG/Fumarate ratio on histone H3K4me3 demethylation (Fig. 6J, O). Since H3K4me3 can directly bind to the *SNCA* promoter region (Fig. 6P), this metabolic correction can inhibit the accumulation of SNCA by reducing the level of H3K4me3. In summary, citrate supplementation corrected the abnormal metabolic flux in the TCA cycle, which, as a beneficial “mito-nuclear” communication signal, inhibited the expression of downstream genes, such as SNCA [27], by targeting and reducing H3K4me3 levels.

## Discussion

Multi-omics datasets from PD patients focused our perspective on mitochondrial damage in PD, showing that mitochondrial function, such as TCA cycle and OXPHOS, were severely impaired in PD in vitro cell model. Moreover, using such models we found that abnormal expression of the MQC system is the molecular basis of mitochondrial dysfunction and that the activation of the MQC process can improve mitochondrial function and alleviate cell damage. ZLN005 and GSK3-IN-3, two MQC activators/inducers, have previously been reported to have beneficial effects on neuronal cells [28, 29]. Here, we further emphasize the protective effects of these two small molecules against MPP^+^-induced neuronal cell toxicity, especially their enhancement of mitochondrial function in in vitro model of PD by activating the MQC system. Although many reports have pointed out that mitochondrial dysfunction is a pathological phenotype associated with cell damage in the progression of PD [3, 4], given the excellent effects of rescue strategies targeting MQC in PD in vitro model, we propose that mitochondrial dysfunction is an early initiator of PD.

To reveal the molecular mechanisms by which mitochondrial dysfunction regulates PD, we performed joint analyses using single-cell transcriptome and serum metabolome datasets from PD patients. Multi-omics analyses enabled us to identify key bottlenecks in TCA cycle metabolism in PD, an important pathway for mitochondrial metabolite turnover, especially in the brain [13]. In particular, IDH3G and MDH2 were severely reduced in the brain of PD patients, which was similar to the expression patterns in the brain of patients with other neurodegenerative diseases [30]. Abnormal expression of TCA cycle metabolic enzymes can cause extensive metabolic remodeling in the cycle [13]. For example, in Early-Onset Severe Encephalopathy, MDH2 mutations cause abnormal accumulation of its substrates malate and fumarate [31]. Here, we show that impaired expression of IDH3G and MDH2, resulting in reduced α-KG levels and upregulated fumarate levels, is an important manifestation of PD TCA cycle disorders and the metabolic basis for mitochondrial-driven PD pathogenesis.

“Mito-nucleus” communication provides an elegant explanation for the crosstalk between mitochondrial metabolism and epigenetics. Many epigenetic enzymes use mitochondrial metabolic intermediates as cofactors to regulate chromatin structure [32]. α-KG and fumarate are active regulators of KDMs [16, 33]. Abnormal changes in their intracellular ratio directly affect the histone demethylation process, which may be achieved by inhibiting the activity of KDMs [34]. It has been reported that H3K4me3 is significantly enriched at the SNCA promoter in the SN of PD patients, and the use of histone demethylase JARID1A can target the reduction of H3K4me3 levels in the SNCA promoter region and inhibit SNCA expression [27]. Here we show that the imbalance of the α-KG/fumarate ratio may lead to the blockade of the conversion of H3K4me3 to H3K4me2 by inhibiting the activity of KDMs, and the targeted reduction of H3K4me3 levels using OICR-9429 can alleviate the abnormal cellular phenotypes associated with PD. Similar to our strategy, the histone acetyltransferase activator YF-2 has also shown effectiveness in the treatment of social isolation in Alzheimer’s disease by targeting histone acetylation modification [35]. These works show the great prospects of drugs targeting epigenetic modifications in the treatment of neurodegenerative diseases.

Currently, about the TCA cycle, supplementation of multiple metabolites including isocitrate [36] and α-KG [37] has been reported to show neuroprotective effects in PD. Based on the metabolic bottleneck of the TCA cycle reported here, we propose citrate as a potential metabolite for the treatment of PD. Actually, citrate can cross the BBB and shows good prospects in improving memory and treating Alzheimer’s disease [38–40]. At the same time, citrate, as α-KG precursor, acts as a metabolic supplement to help increase α-KG levels. More importantly, citrate can also act as a metabolic activator to enhance the activity of MDH2 in a high malate environment, promoting the conversion of fumarate metabolite malate to oxaloacetate [41], which reduces the accumulation of its substrate fumarate. Taken together, the multiple effects of citrate help reduce the α-KG/Fumarate ratio and correct abnormal TCA cycle flux, making it a very promising natural candidate for the treatment of PD. Previous reports have highlighted SNCA accumulation as an upstream event that triggers mitochondrial damage [42]. Here, our work emphasizes mitochondrial damage as an early factor in the pathogenesis of PD, and SNCA accumulation as a downstream molecular event that is finely regulated by α-KG/Fumarate ratio and H3K4me3 levels. These works reflect the complex crosstalk in the pathogenesis of PD and provide a more comprehensive perspective for the development of therapeutic strategies.

A potential limitation of this study is that we used a subacute MPTP-induced PD model to explore the pathogenesis of PD, which may not adequately reflect the chronic progression of PD. Although citrate rescue showed an exciting effect in the MPTP model, long-term safety evaluation of citrate supplementation in a chronic model is still needed.

**Fig. 7:**
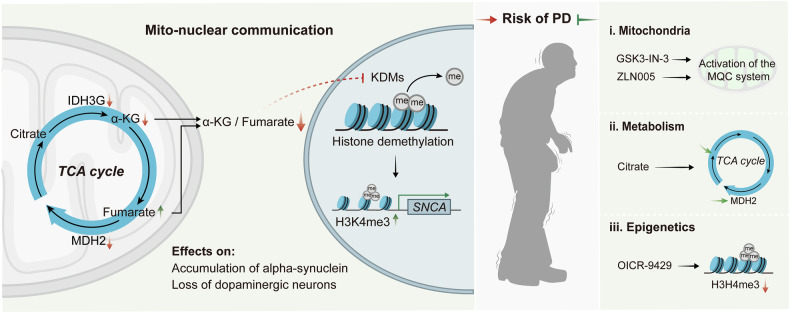
Schematic diagram of the molecular mechanism of TCA cycle disorder promoting PD and related treatment strategies.

In summary, the present study revealed that epigenetic changes driven by mitochondrial TCA metabolic remodeling are closely related to pathophysiology of PD (Fig. 7). Here, we have established a paradigm here that emphasizes the connection between mitochondrial metabolism, epigenetic modification and gene expression regulation through “mito-nuclear” communication. Based on this paradigm, we can try to develop therapeutic strategies targeting the expression regulation of terminal pathogenic genes or anti-disease genes at any level of mitochondrial function, metabolite levels, and chromatin modification, which is not limited to the treatment of PD.

## Materials and methods

### Animal model

Animal models were established using C57BL/6J mice. Mice were maintained in an appropriate environment with free access to food and water.

1-methyl-4-phenyl-1, 2, 3, 6-tetrahydropyridine (MPTP)-induced PD and the citrate supplementation models were developed as previously described [21]. The detailed experimental plans are shown in Fig. 6A. Briefly, mice (8 weeks old) received 12 days of daily drinking water containing citrate (Sigma; 1% w/v) and intraperitoneal injection (i. p.) of MPTP (MedChemExpress, HY-15608; 30 mg/kg body weight) or vehicle treatment for 8th to 12th days; the behavioral teste and sampling were performed three days after the last injection. Citrate was dissolved in drinking water, with pH adjusted to 7.3–7.4 by addition of sodium hydroxide. Midbrain, liver, kidney and serum were immediately collected after the behavioral tests. Some mice were transcardial perfused with cold phosphate-buffered saline (PBS) or PBS containing 4% paraformaldehyde (PFA), and the brain tissues for immunofluorescence staining extracted.

### Cell culture and drug treatment

Human neuroblastoma SH-SY5Y cells obtained from Procell Life Science & Technology Co., Ltd (CL-0208, Wuhan, China) were cultured in MEM/F12 Medium (Procell, Wuhan, China) mixed with 10% fetal bovine serum (FBS, Gibco, USA) and 1% penicillin/streptomycin at 37 °C with 5% CO_2_. To establish an in vitro cell model of PD [16], SH-SY5Y cells were treated for 24 h with 1 μM MPP (MedChemExpress, HY-W008719) and various small molecules purchased from MedChemExpress as follows: 1 μM ZLN005, a potent activator of peroxisome proliferator-activated receptor-γ coactivator-1α (PGC-1α), 1 μM GSK3-IN-3, a mitochondrial autophagy inducer and GSK-3 inhibitor, 0.1 μM, 1 μM or 10 μM CPI-455, a specific KDM5 inhibitor, 0.1 μM OICR-9429, a potent and selective antagonist of the interaction of WDR5 (WD repeat domain 5) with peptide regions of MLL (Mixed Lineage Leukemia) and Histone 3.

### Transcriptome data acquisition

We obtained four human brain datasets from Gene Expression Omnibus (GEO, https://www.ncbi.nlm.nih.gov/GEO/), including one bulk RNA sequencing (GSE205450) and three single-cell sequencing data (GSE157783, GSE126836 and GSE140231). GSE205450 contains 150 samples from caudate (CAU) and putamen (PUT), including 80 normal group and 70 PD group, all data were included in the analysis [43]. GSE157783 contains data from 5 PD cases and 6 normal midbrain tissues, all data were included in the analysis [44]. GSE126836 contains single-cell transcriptomic data of 7 human substantia nigra, but c5828 and c5840 were excluded from the analysis because from brain diseases [45]. GSE140231 contains single-cell data of 7 human substantia nigra, but N3 was excluded from the analysis because from disease [24]. Supplementary Table 1 provided detailed information of bioinformatic analysis.

### Single-cell transcriptome data processing and integration

All data were processed, integrated, and visualized using Seurat (R package, v4.4.0) [25]. Since the data came from different laboratories and were provided in different formats, we adopted different quality control strategies. For GSE157783 data, we used only cells with nFeature_RNA > 200, nCount_RNA > 1000, and ribosomal gene percentage <0.05. For GSE126836 data, we only used cells with 10,000 > nCount_RNA > 1000 and mitochondrial gene percentage <1. For GSE140231 data, we only used cells with 10,000 > nCount_RNA > 1000 and mitochondrial gene percentage <1.5. Next, to remove the batch effect, we used the CCA algorithm, *FindIntegrationAnchors* function to identify anchors between different datasetsand the *IntegrateData* function for integration. The first 10 PCs were used for dimensionality reduction and resolution = 0.3 for visualization.

### Cell type annotation for single-cell transcriptome data

We used classic markers to distinguish different cell types of the nervous system for single-cell transcriptome data. Specifically, we used *MOBP* and *MOG* to identify oligodendrocytes [46], *VCAN* to identify oligodendrocyte precursor cells (OPCs) [47], *AQP4* and *GFAP* to identify astrocytes [48], *GAD1* and *GAD2* to identify neuronal [49], *CD74* to identify microglia [50], *EGFL7 and GLDN5* to identify endothelial [51], and finally, *PDGFRB* to identify pericytes [52].

### Identification of gene with altered expression levels

Due to the differences in bulk RNA sequencing data and single-cell transcriptome data formats and processing software, we used different screening thresholds to identify differentially expressed genes (DEGs). For bulk RNA sequencing data, DESeq2 (R package, v1.42.1) was used and the adjusted *p value* < 0.05 and the absolute value of log2FoldChang > 0.5 was considered as the constraint for identification [53]. For single-cell transcriptome data, Seurat’s *FindMarkers* function was selected to pick up DEGs, and the adjusted *p value* < 0.05 and the absolute value of logFoldChang > 0.5 used as the constraint for identification [25]. The volcano plot was employed to display the distribution characteristics of DEGs, and the heatmap to display the expression patterns of DEGs.

### Acquisition of genes related to mitochondrial function

In order to study the genes involved in mitochondrial disfunctions associated to PD, we used the MitoCarta3.0 database (https://www.broadinstitute.org/mitocarta/mitocarta30-inventory-mammalian-mitochondrial-proteins-and-pathways, Get Date: 2024.05.20) to obtain mitochondrial-related genes.

### Function enrichment analysis

To explore the functions of DEGs, we performed Gene Ontology (GO), Kyoto Encyclopedia of Genes and Genomes (KEGG) enrichment analysis and GSEA using cluster Profiler (R package, v4.10.1) [54]. Briefly, for GO or KEGG, the DEGs list was taken as input, org.Hs.eg.db (R package, v3.18.0) was used as the annotation database, *p-value* correction was performed using B&H methods, and FDR < 0.05 was considered significantly enriched. For GSEA, genes were ranked from largest to smallest and then checked against GO or KEGG pathway gene list as a background gene set, and *p-value* < 0.05 was considered significantly enriched.

### Cell–cell communications analysis

Intercellular communications through ligand-receptor interactions are involved in the regulation of multiple physiological processes and plays an integral role in disease progression [55]. CellChat (R package, v1.6.1) was used to study the cell communication between different cell types in two different states [56]. We used bar graphs and network diagrams to show the intensity and frequency of cell communication between different cell types, and further used stacked bar graphs to visualize the relative information flow of different signaling pathways in different states.

### Analysis of serum metabolome

To investigate the metabolic signature in PD, we collected serum metabolome data from early and late-stage PD [57]. We used *p value* < 0.05 and the absolute value of log2FoldChang > 0.2 as thresholds to identify differential metabolites and volcano plots for visualization.

### Behavioral tests

To assess motor performance and motor coordination, pole and inverted grid tests were used, respectively [21, 58]. Briefly, in pole test, mice were placed on top of a vertically placed wooden pole 50 cm long and 80 mm in diameter. The total time the mice spent climbing from the top to the bottom of the pole were measured. In inverted grid test, mice were placed in the center of the grid (metal mesh, 40 cm^2^ with 0.5 cm^2^ squares) with a surrounding wall, and the grid rotated by 180°. The time the mouse remained on the grid (until the mouse fell) was recorded, with a maximum of 150 s for the test. Mice undergo three learning sessions of behavioral tests before model was established.

### Cell viability

Cells were seeded in a 96-well microplate (1 × 10^4^ cells/well) and cultured for 6, 12, 24 or 48 h. After treatment with MPP, ZLN005, GSK3-IN-3 and OICR-9429, cells were incubated with Cell Counting Kit-8 solution (Abbkine, Wuhan, China, 1:10 in MEM/F12) at 37 °C for 3 h under darkness, and the optical absorbance at 450 nm was measured using a microplate reader (BioTek Cytation, Agilent).

### Measurement of ATP levels and MMP

Cell ATP levels were analyzed using the ATP Assay Kit (Beyotime, S0026, Shanghai, China) according to the manufacturer’s protocol. Chemiluminescence was measured using a microplate reader (BioTek Cytation, Agilent), and the results were expressed as a percentage of the control.

MMP was measured using the MMP Assay Kit with TMRE (Beyotime, C2001S, Shanghai, China) according to the manufacturer’s instructions. After incubation with TMRE staining buffer, the samples were detected by flow cytometry (BD Calibur, CA, USA). Finally, the results were analyzed using FlowJo_v.10 software.

### Reverse transcription quantitative polymerase chain reaction (RT-qPCR)

Total RNA from cultured cells were extracted using a SPARKeasy Improved Tissue/cell RNA kit (Sparkjade, AC0202, Shandong, China), and reverse-transcribed using a SPARKscript II RT plus kit (With gDNA Eraser) (Sparkjade, AG0304) according to the manufacturer’s instructions. cDNA were then mixed with SYBR Premix Ex Taq™ II (Vazyme, Q711-02, Nanjing, China) and gene-specific primers for RT-qPCR in a CFX96 Real-Time System (Bio-Rad Laboratories, CA, USA). A relative quantity was calculated using the 2^−ΔΔCT^ methods. Primer sequences used for qPCR were listed in Supplementary Table 2.

### Enzyme-linked immunosorbent assay (ELISA)

The concentrations of α-KG, Fumarate and Citrate in the cell lysate or serum were measured using enzyme-linked immunosorbent assay kits (Fankew, Shanghai, China) according to the manufacturers’ instructions.

### Western blotting (WB)

Western blot was performed according to the standard protocol [59–61]. The membranes were incubated with primary antibodies: TOMM20 (1:1000; #ab186735, Abcam), PGC1-α (1:1000; No. 381615, ZEN BIO), NRF2 (1:1000; No. 380773, ZEN BIO), TFAM (1:1000; #A3173, ABclonal), MFN1 (1:1000; #A9880, ABclonal), MFN2 (1:1000; #A12771, ABclonal), OPA1 (1:1000; #A9833, ABclonal), DRP1 (1:1000; #ab184247, Abcam), MFF (1:1000; No. R389288, ZEN BIO), PINK1 (1:1000; #DF7742, Affinity Biosciences), PARKIN (1:1000; #A0968, ABclonal), LC3B (1:1000; #ab51520, Abcam), MDH2 (1:1000; #A13516, ABclonal), IDH3G (1:1000; No. 121734, ZEN BIO), OGDHL (1:1000; #A15475, ABclonal), H3K4me2 (1:1000; #A2356, ABclonal), H3K4me3 (1:1000; #A2357, ABclonal), H3 (1:1000; #A17562, ABclonal), SNCA (1:1000; #CY1490, Abways), GAPDH (1:5000; #AF7021, Affinity Biosciences) overnight at 4 °C. After washing, the membranes were incubated with the horseradish peroxidase-labeled Goat Anti-Rabbit immunoglobulin G (1:5000; #A0208, Beyotime) for 1 h, and protein bands visualized using Tanon 5200 multi-imager (Tanon Science & Technology, China).

### Immunofluorescence staining (IF)

Brains were fixed in 4% PFA at 4 °C for 24 h. Then, samples were transferred sequentially to PBS solutions with 20% or 30% sucrose (w/v), and stored at 4 °C for at least 24 h until the slicing procedure. Serial 20 μm coronal sections were collected from frozen brains and mounted on slides for subsequent histological procedures. Briefly, sections were blocked with 5% bovine serum albumin and then incubated with antibodies against TH (1:200; No. R381285, ZEN BIO) at 4 °C overnight. After washing, the slides were incubated with goat anti-rabbit Alexa Fluor 555 (1:200; #ab150078, Abcam) for 1 h at 37 °C and counterstained with Hoechst 33342 (Beyotime, C1022, Shanghai, China) for 6 min at room temperature (RT). Finally, the slides were washed in PBS and observed under a fluorescence microscope (Olympus BX51, Japan). TH levels were quantified by fluorescence intensity analysis. All images used for fluorescence intensity analysis were captured at the same exposure time. TH fluorescence intensities from four regularly spaced 20 μm sections were acquired from each brain (*n* = 3 animals per group). The TH fluorescence intensity of each sample was averaged and expressed as a percentage of the control.

### TUNEL staining

Cells were fixed in 4% PFA at RT for 30 min before transferring onto a glass slide and dried onto a hotplate. After samples washing with PBS, staining was performed using the TUNEL BrightRed Apoptosis Detection Kit (Vazyme, A113-03, Nanjing, China) according to the manufacturers’ protocols. The level of apoptosis was quantified as the number of TUNEL-positive cells as a percentage of the total number of cells in the same field of view and expressed as a percentage of the control.

### Cleavage under targets & release using nuclease and qPCR detection (CUT&RUN-qPCR)

Midbrains from 9-week-old mice were collected and treated with 2 mg/ml collagenase at 37 °C for 20 min to obtain single-cell suspensions. Cell counting and viability tests were performed to ensure that there were ~500,000 cells in each group (IgG group and H3K4me3 group) and that cell viability was normal. CUT&RUN assays were performed using the Hyperactive pG-MNase CUT&RUN PCR/qPCR Assay Kit (Vazyme, HD-101) according to the manufacturer’s instructions. Briefly, cells were incubated with ConA Beads Pro at RT for 10 min, beads were collected, antibodies were added, and incubated overnight at 4 °C. Magnetic beads were collected and incubated with pG-MNase enzyme at 4 °C for 1 h. Subsequently, CaCl_2_ was added to the samples to activate DNA fragmentation. After incubation on ice for 2 h, stop solution (containing DNA Spike for normalization and calibration of qPCR data) was added, and DNA was extracted from the chromatin enrichment product after incubation at 37 °C for 20 min for qPCR detection. The Light Cycler 480 II apparatus (Roche, Germany) was used to perform qPCR utilizing SYBR Premix Ex Taq™ II (Vazyme, Q711-02). The antibodies used in CUT&RUN are as follows: H3K4me3 (1:30; #A22146, ABclonal), control rabbit IgG (1:30; #A7016, Beyotime). The primer sequences of the *Snca* promoter region used in CUT&RUN-qPCR are as follows:

F: 5′-CCCTCTCTGTAGGGTGAGGAG-3′;

R: 5′- TGATAGTGGCAGGGTTTTGATGG-3′.

### Statistical analysis

All data are presented as mean ± standard error of mean (SEM). The Student’s *t* test was used for comparison between two groups. One-way analysis of variance (ANOVA) was used to assess differences among multiple groups. Differences were considered statistically significant at *P < 0.05, **P < 0.01, and ***P < 0.001.

## Supplementary information

Supplementary data

Original western blots

## Data Availability

All data are available upon reasonable request.
