# Supplementary material for: Mitochondrial dysfunction-mediated metabolic remodeling of TCA cycle promotes Parkinson’s disease through inhibition of H3K4me3 demethylation
Source: Cell Death Discov. 2025 Jul 29;11:351. doi: 10.1038/s41420-025-02651-1 (PMC12307738; doi:10.1038/s41420-025-02651-1)
Supplement: Supplementary file 1 — Supplementary data [file 41420_2025_2651_MOESM1_ESM.docx]

**Mitochondrial dysfunction-mediated metabolic remodeling of TCA cycle promotes Parkinson's disease through inhibition of H3K4me3 demethylation**

Xiaoyuan Zhang^#^, Fali Zhang^#^, Yue Zeng, Aiying Li, Jiamao Yan, Pei Li, Kexin Qin, Teng Zhang, Jiaojiao Huang, Minghui Zhao, Massimo De Felici, Yang Zhou* & Wei Shen*

**Author information**

^#^These authors contributed equally: Xiaoyuan Zhang, Fali Zhang.

Authors and Affiliations

**College of Animal Science and Technology, Qingdao Agricultural University, Qingdao, 266109, China**

Xiaoyuan Zhang, Fali Zhang, Yue Zeng, Aiying Li, Jiamao Yan, Pei Li, Kexin Qin, Jiaojiao Huang, Minghui Zhao & Wei Shen

**State Key Laboratory of Reproductive Regulation and Breeding of Grassland Livestock (R2BGL), College of Life Sciences, Inner Mongolia University, Hohhot, 010070, China**

Xiaoyuan Zhang, Jiamao Yan, Teng Zhang & Yang Zhou

**Department of Biomedicine and Prevention,** **University of Rome Tor Vergata,** **Rome** **00133,** **Italy.**

Massimo De Felici

Corresponding authors

*Correspondence to Wei Shen or Yang Zhou.

Correspondence and reprint requests to:

Prof. Wei Shen; E-mail: wshen@qau.edu.cn

Prof. Yang Zhou; E-mail: zhouyang106@126.com

**Supplementary figures and figure legends**


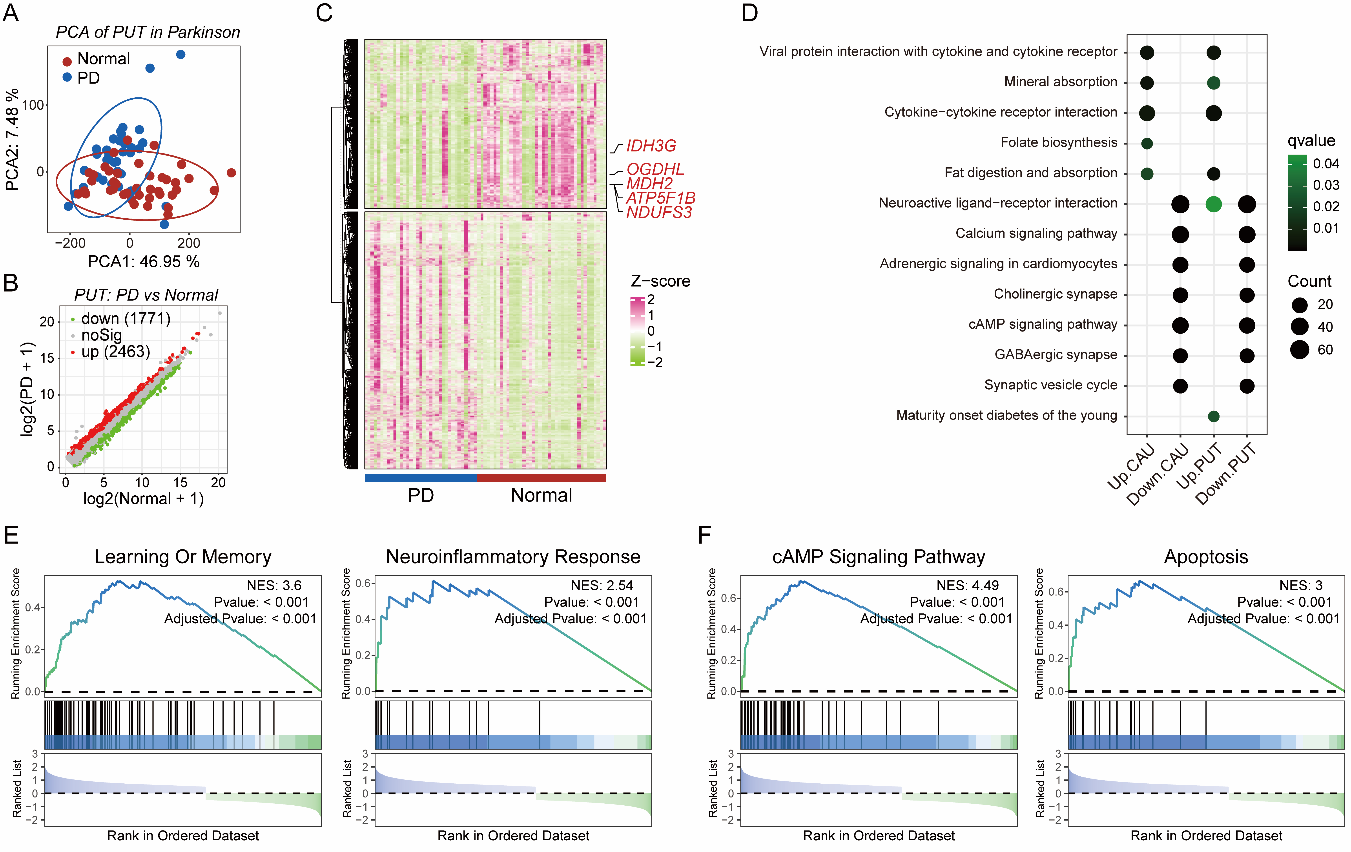


**Fig. S1 Transcriptome analysis of PUT in PD**

**A, B** Principal component analysis (**A**) and DEGs (**B**) of transcriptome data of PUT from normal groups and PD groups. **C** Heat map shows the expression patterns of DEGs of the transcriptome data of PUT in the normal group and PD group, and some genes of interest are listed on the right. **D** GO analysis of DEGs based on CAU and PUT transcriptome data. **E, F** GSEA analysis of PUT transcriptome data based on the relevant GO term gene list (**E**) and KEGG pathway gene list (**F**).


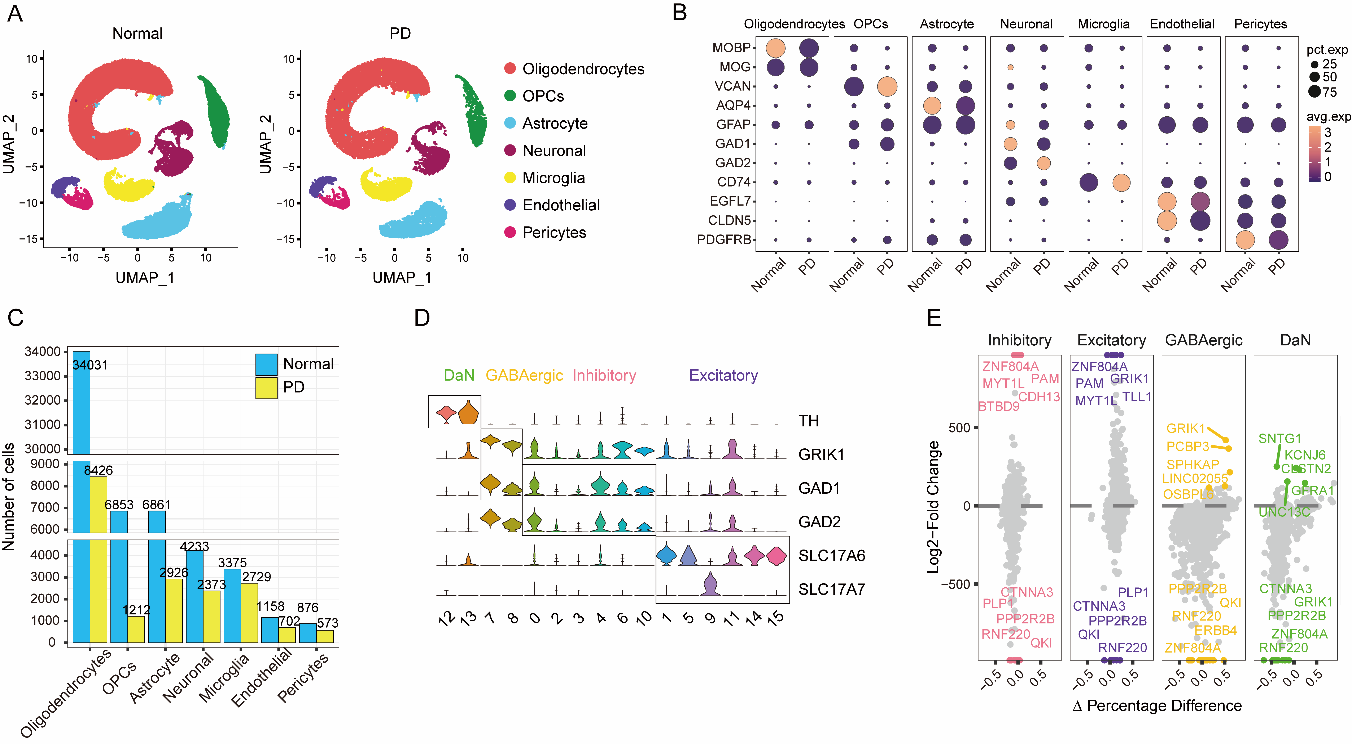


**Fig. S2 Single-cell transcriptome analysis of the midbrain in PD**

**A** UMAP plot shows the distribution characteristics of each nervous tissue cell type in the normal group (left) and PD group (right). **B** Dot plot showing the marker gene expression of seven identified cell types. **C** Number of seven different nervous tissue cell types in the two investigated groups. **D** Violin plot showing the marker gene expression of four neuronal cell subtypes in each cluster. **E** The top five DEGs for each neuronal cell subtype.

**Supplementary Table 1. The detail information of data collection.**

| **GEO ID** | **Dataset Type** | **Organism** | **Main Analysis** | **Related Figures** |
| --- | --- | --- | --- | --- |
| GSE205450 | Bulk RNA-seq | Human,  Striatum | - Identification of DEGs - Function enrichment | Fig1, Fig2, FigS1 |
| GSE157783 | Single-cell RNA-seq | Human,  Midbrain | - Identification of Neuronal cells - Function enrichment | Fig4, FigS2 |
| GSE126836 | Single-cell RNA-seq | Human,  Substantia nigra | - Identification of Neuronal cells - Function enrichment | Fig4, FigS2 |
| GSE140231 | Single-cell RNA-seq | Human,  Substantia nigra | - Identification of Neuronal cells - Function enrichment | Fig4, FigS2 |

**For single-cell detail information.**

The number of different cell types detected in different sample

| **Celltype** | **Midbrain** (GSE157783) | **Substantia nigra** (GSE126836, GSE140231) |
| --- | --- | --- |
| Oligodendrocytes | 21005 | 21452 |
| Neuronal | 5312 | 1294 |
| Endothelial | 1691 | 169 |
| Astrocyte | 5374 | 4413 |
| OPCs | 2479 | 5316 |
| Microglia | 3952 | 2152 |
| Pericytes | 1227 | 222 |

The number of different cell types detected in different group

| **Celltype** | **Normal** | **PD** |
| --- | --- | --- |
| Oligodendrocytes | 34031 | 8426 |
| Neuronal | 4233 | 2373 |
| Endothelial | 1158 | 702 |
| Astrocyte | 6861 | 2926 |
| OPCs | 6853 | 1212 |
| Microglia | 1158 | 702 |
| Pericytes | 876 | 573 |

**Supplementary Table 2. The qPCR primer sequence used in this study.**

| **Gene symbol** | **Primer sequences** |
| --- | --- |
| *MDH2* | *MDH2* (F): AGCACCGGAAGAGTCGCT  *MDH2* (R): CTTCCCCAGCTGTTCTCTGAGG |
| *IDH3G* | *IDH3G* (F): GGAGGCTCTCACTTTCCGTC  *IDH3G* (R): AGCGGACGGAGGAATTGTTT |
| *OGDHL* | *OGDHL* (F): TCGAGGTGAGCCAGCTCTAT  *OGDHL* (R): GCACCTGGGTACTTCTCTGC |
| *NDUFS3* | *NDUFS3* (F): CGGCAGAACCGTTTTGAG  *NDUFS3* (R): TCAATGGGTGTCAGCTCATC |
| *COX6B2* | *COX6B2* (F): CTAACGCCCTCACCATTT  *COX6B2* (R): TCGCATCATTGTACTCCAG |
| *ATP5F1B* | *ATP5F1B* (F): ACCTCGGTGCAGGCTATCTA  *ATP5F1B* (R): AATAGCCCGGGACAACACAG |
| *GAPDH* | *GAPDH* (F): ACCCAGAAGACTGTGGATGG  *GAPDH* (R): CACATTGGGGGTAGGAACAC |

**Supplementary Table 3. List of tissue-conserved mitochondrial-related DEGs.**

| **Candidate mitochondrial function genes** | |
| --- | --- |
| HIBCH | PCCB |
| AGPAT4 | OGDHL |
| MRPS6 | ATAD1 |
| SLC25A12 | ADCY10 |
| GDAP1 | NT5DC2 |
| TOMM70 | PRELID2 |
| GLS | TOMM20 |
| SLC25A23 | FASN |
| SLC25A27 | HSPD1 |
| MRPS5 | PCBD2 |
| KYAT3 | ACCS |
| ATP5F1C | TOMM34 |
| DNA2 | ARF5 |
| CHPT1 | ATP5F1B |
| ME3 | MTCH2 |
| TRAP1 | PDP1 |
| SLC25A13 | PDE2A |
| GLS2 | MDH2 |
| PREPL | OCIAD2 |
| COX19 | GLOD4 |
| MGST1 | SLC25A14 |
| CHDH |  |
