# Supplementary material for: Mitochondrial dysfunction-mediated metabolic remodeling of TCA cycle promotes Parkinson’s disease through inhibition of H3K4me3 demethylation
Source: Cell Death Discov. 2025 Jul 29;11:351. doi: 10.1038/s41420-025-02651-1 (PMC12307738; doi:10.1038/s41420-025-02651-1)
Supplement: Supplementary file 2 — Original western blots [file 41420_2025_2651_MOESM2_ESM.docx]

GAPDH TOMM20

Fig. 2J





 GAPDH PARKIN

Fig. 3B







 DRP1 MFN1

Fig. 3B



 TFAM LC3B II/LC3B I

Fig. 3B

PGC1-α OPA1










 Fig. 3B



 MFF NRF2

Fig. 3B



 MFN2 PINK1

Fig. 3B

Fig. 4H GAPDH OGDHL







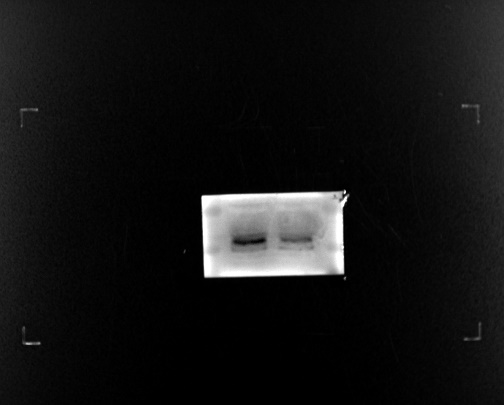


Fig. 4H IDH3G MDH2





 GAPDH H3

Fig. 5F

H3K4me3 H3K4me2

Fig.



5F





 GAPDH H3

Fig. 5G





 H3K4me3 H3K4me2







Fig. 5G

GAPDH H3

Fig. 5J



 H3K4me3 H3K4me2

Fig. 5J

Fig. 6J GAPDH H3












 H3K4me2 H3K4me3

Fig. 6J

SNCA

Fig. 6J
